# Response Characteristics of Pressure-Sensitive Conductive Elastomer Sensors Using OFC Electrode with Triangular Wave Concavo-Convex Surfaces

**DOI:** 10.3390/s24072349

**Published:** 2024-04-07

**Authors:** Takeru Katagiri, Sogo Kodama, Kotaro Kawahara, Kazuki Umemoto, Takanori Miyoshi, Tadachika Nakayama

**Affiliations:** 1 Department of Science of Technology Innovation, Nagaoka University of Technology, 1603-1 Kamitomioka, Nagaoka, Niigata 940-2188, Japan; 2 Department of Mechanical Engineering, Nagaoka University of Technology, 1603-1 Kamitomioka, Nagaoka, Niigata 940-2188, Japanumemoto@u-fukui.ac.jp (K.U.); 3 INABA RUBBER Co., Ltd., 3-3-15 Kyomachibori, Nishi, Osaka 550-0003, Japan; 4 Department of System Safety, Nagaoka University of Technology, 1603-1 Kamitomioka, Nagaoka, Niigata 940-2188, Japan

**Keywords:** conductive elastomer, conductive rubber, pressure sensor, piezoresistive sensor, elastomer, composite

## Abstract

The sensor response of pressure-sensitive conductive elastomers using polymeric materials can be adjusted by altering the type and quantity of fillers used during manufacturing. Another method involves modifying the surface shape of the elastomer. This study investigates the sensor response by altering the surface shape of an electrode using a readily available pressure-sensitive conductive elastomer. By employing an oxygen-free copper electrode with a flat surface (with surface roughness parameters Ra = 0.064 μm and Rz = 0.564 μm) as a baseline, we examined the sensor system’s characteristics. Electrodes were fabricated with triangular wave concavo-convex surfaces, featuring tip angles of 60, 90, and 120°. Improved sensor responses were observed with electrodes having tip angles of 60 and 90°. Additionally, even with varying conductive properties of elastomers, the conductance of the elastomer sensor increased similarly when using an electrode with a 90° tip angle. This study demonstrates the potential for expanding the applications of conductive elastomer sensors, highlighting the noteworthy improvement in sensor response and performance achieved by altering the surface shape of electrodes used with commercially available conductive elastomers.

## 1. Introduction

Functional materials incorporating polymer materials and conductive fillers encompass a wide range of applications, including catalysts [1], actuators [2], electromagnetic shields [3,4], gas sensors [5], temperature sensors [6], and humidity sensors [7]. Force sensors, being flexible, can conform to curved surfaces, enabling their attachment to the human body for utilization as IoT devices, particularly in biological monitoring [6]. These force sensors, using polymeric materials, exhibit changes in capacitance [8,9,10,11,12,13] and resistance as sensor responses. Additionally, beyond their pressure-sensitive functionalities, certain devices demonstrate self-repair capabilities [14] and self-power generation [7,15].

In the realm of force sensor applications, proposals have been made for object recognition using machine learning [16,17,18] and surface roughness identification [19,20]. A case study using a commercially available conductive elastomer composite as a sensor for machine learning [21,22] reported a 92.73% correct response rate for object recognition. Furthermore, a slip-detectable robotic hand using a conductive elastomer composite was documented in [23]. Conductive elastomers serve as versatile force-measuring instruments, providing pressure-sensitive functionality across both flat and curved surfaces, making them applicable in various fields. Essentially, the responses of elastomer sensors directly influence the efficacy of classification methods used in machine learning. Integrating these sensors onto robots can considerably affect their controllability, underscoring the importance of enhancing conductive elastomer performance.

Criteria for evaluating the functionality of a force sensor include sensitivity, linearity, variability of sensor response, and rise time. Methods aimed at improving these criteria involve employing mixed fillers and modifying the surface shape of elastomers. The pressure-sensitive electrical conduction mechanism in piezoresistive elastomers containing electrically conductive fillers has been elucidated, citing chain formations [24] and the tunneling effects of electrically conductive particles within the elastomer [25,26,27,28,29,30]. The content of conductive filler is constrained by the percolation threshold [31,32], necessitating adjustments in filler content or the utilization of multiple filler types to improve pressure-sensitive characteristics. In addition to utilizing combined conductive fillers, internal design methods involve controlling the arrangement of these fillers through magnetic and electric fields [33,34].

Methods for alternating sensor response include modifying the filler composition within elastomers and adjusting the surface shape of the elastomer [13,35,36,37,38,39]. An elastomer with an uneven surface can expand the area of non-contact surfaces when the elastomer and electrode, or elastomers themselves, are in contact under no-load conditions, thereby increasing insulation properties. When an external force is applied in this condition, it alters the local contact area between the elastomer and electrode (or between the elastomers). This facilitates the detection of changes in electrical signals, thereby enabling the introduction of a pressure-sensitive function to a material that initially lacks such capability or detecting minute external forces.

In addition to altering the sensor response by modifying the filler composition within the elastomer or changing its surface shape, we propose a method to enhance sensor response by changing the surface shape of the electrode interfacing with the elastomer. If the sensor response can be altered by varying the surface shape of the electrode paired with the elastomer, it allows for the utilization of existing elastomers without necessitating material change. Therefore, sensor design can be tailored to specific applications without altering the elastomer material, offering versatility. Additionally, unlike manufacturing techniques that intentionally create uneven elastomer surfaces, our approach allows for modification in sensor response characteristics even after polymer material hardening, eliminating the need to change existing elastomer manufacturing processes. The key advantage of this method lies in its simplicity, as it enhances sensor response using readily available conductive elastomers. By enhancing the sensitivity and responsiveness of commercially available conductive elastomer sensors, they become viable for applications in machine learning or robot control applications, thereby expanding their utility and functionality in current settings. Quasi-static characteristics of the proposed sensor were observed when electrodes featured flat and triangular wave concavo-convex surfaces. Moreover, dynamic sensor characteristics were assessed by subjecting the elastomer sensor to step-like and impulse-like external forces.

Subsequent in-depth experimental results will demonstrate how the surface shape of the electrode affects sensor response, highlighting the effectiveness of triangular wave concavo-convex surfaces in improving sensor performance across several aspects.

## 2. Materials and Methods

### 2.1. Material Selection

Three types of conductive elastomers, sourced from INABA RUBBER Co., Ltd., Kyomachibori, Nishi, Osaka each exhibiting distinct conductive properties, were used in the study. By placing these conductive elastomers onto electrodes, they functioned as pressure-sensitive sensors, with their electrical properties showing improvements under increased pressure. The manufacturer provided specifications for each elastomer variant, marketed as high-sensitivity, middle-sensitivity, and low-sensitivity elastomers. Each elastomer had a thickness of 0.5 mm and the length and width were each 20 mm. We utilized a high-sensitivity elastomer to analyze the effects of different electrode surface shapes on the quasi-static characteristics (Section 3.1) and dynamic characteristics (Section 3.3). Middle- and low-sensitivity elastomers were used to investigate whether differences in electrode surface shape had an effect regardless of the conductive properties of the conductive elastomer (Section 3.2).

**Figure 1:**
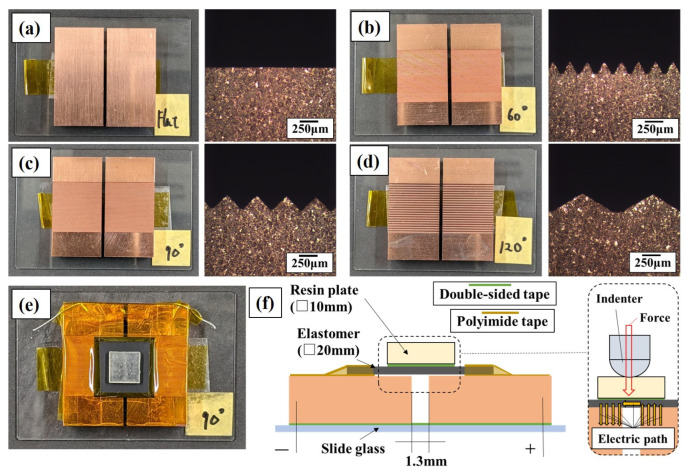
Digital photographs of (**a**) Flat electrode; (**b**) 60° electrode; (**c**) 90° electrode; (**d**) 120° electrode; (**e**) Elastomer sensor; (**f**) Schematic cross section of elastomer sensor.

Figure 1 shows the electrodes used with the conductive elastomers. Particularly, Figure 1a showcases oxygen-free copper (OFC) characterized by a surface roughness of Ra = 0.064 μm and Rz = 0.564 μm, referred to as the flat electrode throughout this study. To compare sensor responses with the flat electrode, three types of electrodes with triangular wave concavo-convex were fabricated. Figure 1b demonstrates a concavo-convex surface featuring a tip angle of 60°, pitch of 0.23 mm, and a peak height of 0.15 mm, denoted as the 60° electrode. Figure 1c describes a concavo-convex surface with a tip angle of 90°, a pitch of 0.40 mm, and a peak height of 0.18 mm, termed as 90° electrode. Finally, Figure 1d presents an electrode with a concavo-convex surface having a tip angle of 120°, pitch of 0.95 mm, and peak height of 0.24 mm, labeled as the 120° electrode. Electrodes with triangular wave concavo-convex surfaces were manufactured by cutting grooves using an engraving cutter on OFC made of the same material, as shown in Figure 1a. The electrode surface area is determined by configuration of the engraving cutter’s blade edge, which offers three different tip shapes: 60, 90, and 120°. Applying these tip shapes to the electrode surface results in a triangular wave concavo-convex configuration, yielding a larger surface area than the flat electrode. It is assumed that there were no flat segments at the tip of the triangular wave-shaped peaks and valleys formed on the electrode surface by the engraving cutter. Thus, the electrode surface exhibits a triangular wave concavo-convex profile mirroring the angle of the engraving cutter’s tip, without any gaps. The calculated surface area of the electrode represents its apparent surface area. Accordingly, the apparent surface areas of the 60, 90, and 120° electrodes are approximately 2, 1.41, and 1.15 times larger, respectively, than that of the flat electrode. It is important to note that electrodes alone are incapable of measuring forces; they must be arranged in the configuration shown in Figure 1e to detect applied forces. This arrangement constitutes what is called an elastomer sensor. Additionally, Figure 1f represents a schematic cross section of the elastomer sensor. Polyimide tape was applied over the electrode to create an insulating barrier. The outer periphery of the elastomer overlapped with this insulating area, while the upper surface of the elastomer was fixed using polyimide tape, effectively sandwiching the elastomer between layers of polyimide tape. A resin plate measuring 10 × 10 mm was positioned atop the elastomer, spanning the electrode. Upon contact with the indenter, the elastomer and electrode made contact, establishing a conductive path between them. Additionally, uniform force was exerted on the conductive elastomer surface by pressing the center of the resin plate. The distance between the electrodes was maintained at 1.3 mm.

### 2.2. Acquisition Method of Quasi-Static Characteristics of Elastomer Sensors

**Figure 2:**
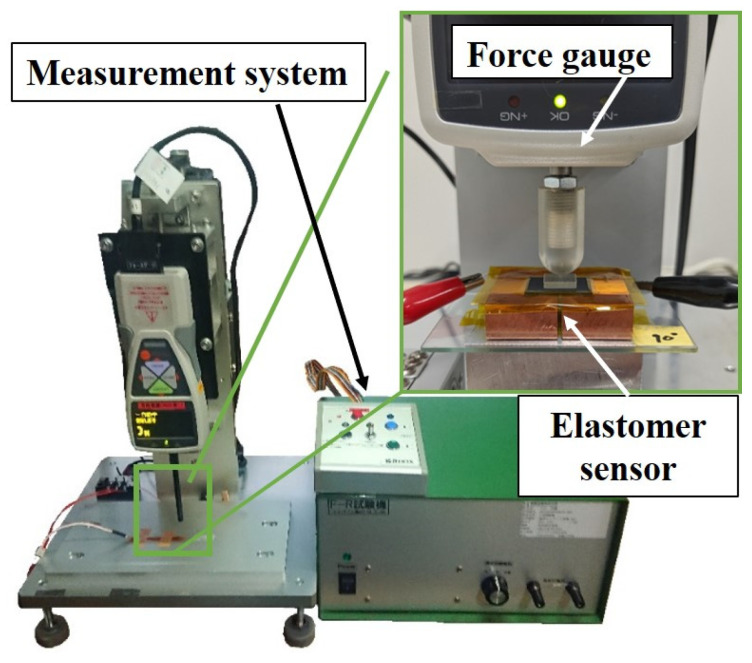
Pressure testing machine for obtaining the quasi-static characteristics.

Several elastomer sensors [21] exhibited the characteristic ongoing sensor response fluctuations even when under a constant load. Similar behavior was observed with the proposed conductive elastomers. Therefore, even when a consistent external force was applied to the elastomer sensor, it did not maintain a steady, unchanging value. The conductance changes observed when a load is applied to the elastomer sensor include the effect of the load alteration and the evolution over time, constituting the sensor’s response. In this study, the characteristics obtained from these conductance changes occurring when an external force is applied to the elastomer sensor at a constant speed are referred to as quasi-static characteristics. Quasi-static characteristics were evaluated using a pressure testing machine (manufactured by INABA RUBBER Co., Ltd., Osaka, Japan: F-R testing machine), as depicted in Figure 2. This involved pressing the center of the sensor’s resin plate with the hemispherical tip of the machine’s indenter, starting from a non-contact position, and applying pressure perpendicular to the elastomer at a speed of 100 cm/min. Simultaneously, the force increases to 30.58 N. Upon reaching the maximum load, the indenter ascends at a rate of 100 cm/min to release the applied pressure, eventually returning the elastomer sensor and indenter to their non-contact states. During the pressurization and depressurization processes, the resistance value undergoes fluctuations as the indenter moves. Subsequently, the obtained resistance value is converted into conductance to assess the conductance change in response to the external force input. The pressurization test is repeated five times, and the mean value and standard deviation are determined. Moreover, a regression line is calculated from the sensor response up to 10 N of compression, and the sensor sensitivity is compared. The slope of this regression line, denoted as sensor sensitivity, is measured in mS/N. This value, along with the conductance acquired in this test, is used to evaluate the dynamic characteristics described in the subsequent subsection. To evaluate dynamic characteristics, a nominal value for the elastomer sensor’s external force is required instead of a steady value, enabling the assessment of conductance changes over time defining the nominal values. Among the conductance values obtained during pressurization up to 10 N, the mean conductance of the elastomer sensor under external forces of 1, 5, and 10 N depicted in serves as the nominal value. Throughout this study, this nominal value is referred to as the nominal conductance value. The evaluation results of the quasi-static characteristics evaluation using this testing device are shown in Section 3.1 and Section 3.2.

### 2.3. Acquisition Method of Dynamic Characteristics of Elastomer Sensors

**Figure 3:**
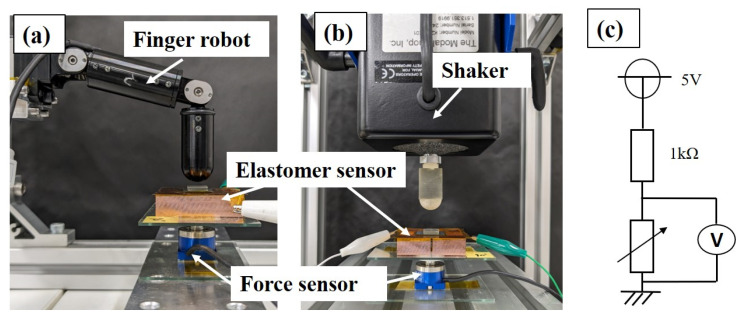
Experimental environment for step-like and periodic impulse-like input: (**a**) Step-like input using a finger robot; (**b**) Periodic impulse-like input using a shaker; (**c**) Sensor circuit for acquiring potential difference of elastomer sensor.

As aforementioned, the response of an elastomer sensor undergoes fluctuations even under a consistently applied load. This ongoing change in sensor response is a crucial characteristic to consider when implementing long-term force control using an elastomer sensor response in a robotic system. Figure 3 shows the experimental setup used to capture the time response of the elastomer sensor. In Figure 3a, the sensor response was captured upon applying a step-like input to the elastomer sensor using a finger robot (manufactured by Harmonic Drive Systems Inc., Tokyo, Japan: Finger module). The measurement start time was designated as the moment when the elastomer sensor’s response reached a nominal conductance value of 10% for each external force recorded during the quasi-static characteristic test. After 300 s from the onset of measurements, the conductance of the elastomer sensor was assessed. The difference between the conductance value of the elastomer sensor after 300 s and its nominal conductance value was defined as the conductance creep amount under a constant load. Additionally, the rise time of the elastomer sensor upon application of a step-like input was evaluated using the nominal conductance value obtained from the quasi-static characteristic test for each external force. A step-like input was administered to the elastomer sensor, and the time taken for the elastomer sensor’s response to transition from 10% to 90% of the nominal conductance was determined. To evaluate variations in conductance and time increase in response to step-like inputs, tests were repeated five times under each external force condition, and the mean and standard deviation were calculated. Furthermore, a shaker (manufactured by The Modal Shop, Inc., Cincinnati, OH, America: Model K2004E01), shown in Figure 3b, was used to capture the elastomer sensor’s response to external forces, such as impulse inputs, administered for extremely brief durations. The elastomer sensor underwent impulse-like inputs facilitated by a shaker. Equipped with an indenter at its tip, the shaker repeatedly transitions from a non-contact state to a contact state, applying a force input perpendicular to the elastomer sensor at a predefined frequency and external force magnitude. Directly beneath the elastomer sensor, a force sensor (manufactured by Nippon Liniax Co., Ltd., Osaka, Japan: MFS20-010) was installed to measure the applied external force. Alignment of sensor waveforms under each experimental condition with the external force waveforms recorded by the force sensor confirmed the equivalence of forces applied to the elastomer sensor. Consequently, management of the external force applied to the elastomer sensor relied on the waveforms captured by the force sensor. Figure 3c shows the sensor circuit used to capture the elastomer sensor’s response. A DC voltage of 5 V was applied with an external resistance of 1 kΩ. The potential difference induced by the elastomer between the electrodes was measured, and resultant voltage changes were converted into conductance variations. For measurements involving step-like input, the sampling period for the potential difference between the elastomer and force sensor was set at 10 kHz. Furthermore, during assessments under impulse-like inputs, the frequency was adjusted to 1 MHz. Simultaneous measurement of the elastomer sensor response and the force sensors was conducted. We evaluated the sensor response under external forces of 1, 5, and 10 N for both step-like and impulse-like inputs. The results regarding the dynamic characteristics are presented in Section 3.3.

## 3. Result

### 3.1. Quasi-Static Characteristics

**Figure 4:**
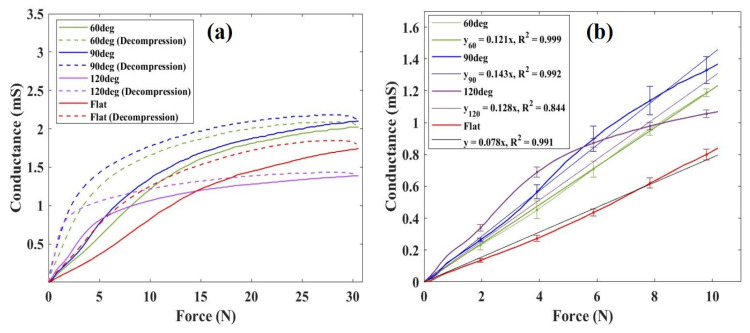
(**a**) Mean value of conductance changes of elastomer sensor due to difference in electrode surface shape; (**b**) Mean value of conductance change, standard deviation per 1.96 N, and regression line when external force is applied up to 10 N.

Figure 4 shows the sensor responses obtained using a highly sensitive elastomer under flat, 60, 90, and 120° surface conditions. Figure 4a depicts the conductance of the sensor when subjected to an external force of 30 N, followed by pressure reduction. Previous studies have reported variations in elastomer sensors’ responses during pressurization and depressurization [25], a phenomenon similarly observed in the conductive elastomers used in this study. Comparative analysis reveals enhanced conductance responses when using the 60° and 90° electrodes compared to the flat electrode. Particularly, under a 30 N load, the mean conductance value with the flat electrode was 1.73 mS, whereas with the electrodes featuring triangular wave concavo-convex surfaces, it measured 2.02 and 2.09 mS for the 60° and 90° configurations, respectively. Notably, under a 30 N load, the conductance improved by 1.16 times with the 60° electrode and 1.21 times with the 90° electrode compared to the flat electrode. Furthermore, using this elastomer sensor, we obtained a highly linear sensor response for loads approximately 10 N, indicating its sensitivity up to this pressure-sensitive range. Illustrated in Figure 4b is the sensor response under external forces up to 10 N, along with its regression line. The sensitivity values were measured at 0.078 and 0.121 for the flat electrode and the 60° electrode, and 0.143 for the 90° electrode. Comparatively, the sensitivity improved by 1.55 and 1.83 times with the 60° and 90° electrodes, respectively, in contrast to the flat electrode. However, upon examination of the standard deviation for each 1.96 N increment, we found that the standard deviation was greater when using the 60° and 90° electrodes than the flat electrode, indicating an increase in variation in the elastomer sensors’ response to pressurization. Interestingly, with the 120° electrode, conductivity did not improve even under a 30 N, and the linear pressure-sensitive range became narrower. Consequently, based on these results, it is evident that under the given experimental conditions, the performance of the elastomer sensor can be improved using the 90° electrode.

### 3.2. Comparison of Sensor Response Using Elastomers with Different Conductive Properties

**Figure 5:**
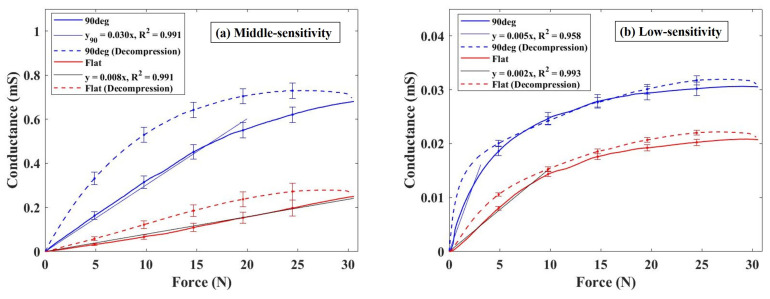
Comparison of sensor response using elastomers with different conductive properties: (**a**) Middle-sensitivity elastomer; (**b**) Low-sensitivity elastomer.

Figure 5 shows the relationship between conductance change and external force magnitude when utilizing two types of conductive elastomers with different conductive properties, tested with both flat electrode and 90° electrode. Figure 5a,b depict the results obtained with medium- and low-sensitivity elastomers, respectively. Across all conductive elastomers with different conductive properties, employing the 90° electrode resulted in enhanced measured conductance compared to using a flat electrode. Figure 5a shows the results for the medium-sensitivity elastomer, revealing a conductance of 0.25 mS with a 30 N load on the flat electrode, while registering 0.68 mS on the 90° electrode, indicating a 2.72 times higher conductance with the 90° electrode. However, employing the 90° electrode narrowed the pressure-sensitive range with linearity compared to the flat electrode. Notably, the coefficient determination value of the regression line remained the same for both electrodes, indicating a sensitivity of 0.008 for the flat electrode under a 30 N load and 0.03 under a 20 N load for the 90° electrode. Moreover, when utilizing the 90° electrode, a significant difference in conductance change between pressurization and depressurization was observed. When using the low-sensitivity elastomer, the conductance measured 0.02 mS under a 30 N force applied to the flat electrode (refer to Figure 5b). Furthermore, when using the 90° electrode, the conductance increased to 0.03 mS, marking a 1.50 times higher reading than with the flat electrode. In the case of low-sensitivity elastomers, the linear pressure-sensitive range narrowed, and a sensitivity of 0.002 was obtained when a force of approximately 10 N was applied to the flat electrode. When employing the 90° electrode, the linear pressure-sensitive range exhibited a narrower profile than that for the flat electrode. Quantitatively, a coefficient of determination of 0.958 was observed, with a sensitivity of 0.005 recorded when applying a 3 N load during the pressurization phase.

These results indicate instances where differences in sensor response between pressurization and depressurization were increased when utilizing the 90° electrode. Additionally, it is evident that this configuration may lead to a reduction in the range of the pressure-sensitive range with linearity. Although this narrowing could potentially reduce sensor performance, it was confirmed that the conductivity between the elastomer and electrode could be improved with the 90° electrode, regardless of the differing conductive properties of the elastomers. Therefore, it is plausible to suggest that the improvement in electrical conductivity resulting from the utilization of a triangular wave concavo-convex surface is not specific to a single elastomer but applicable to several elastomers.

### 3.3. Dynamic Characteristics

#### 3.3.1. Sensor Response Evaluation by Step-like Inputs

**Figure 6:**
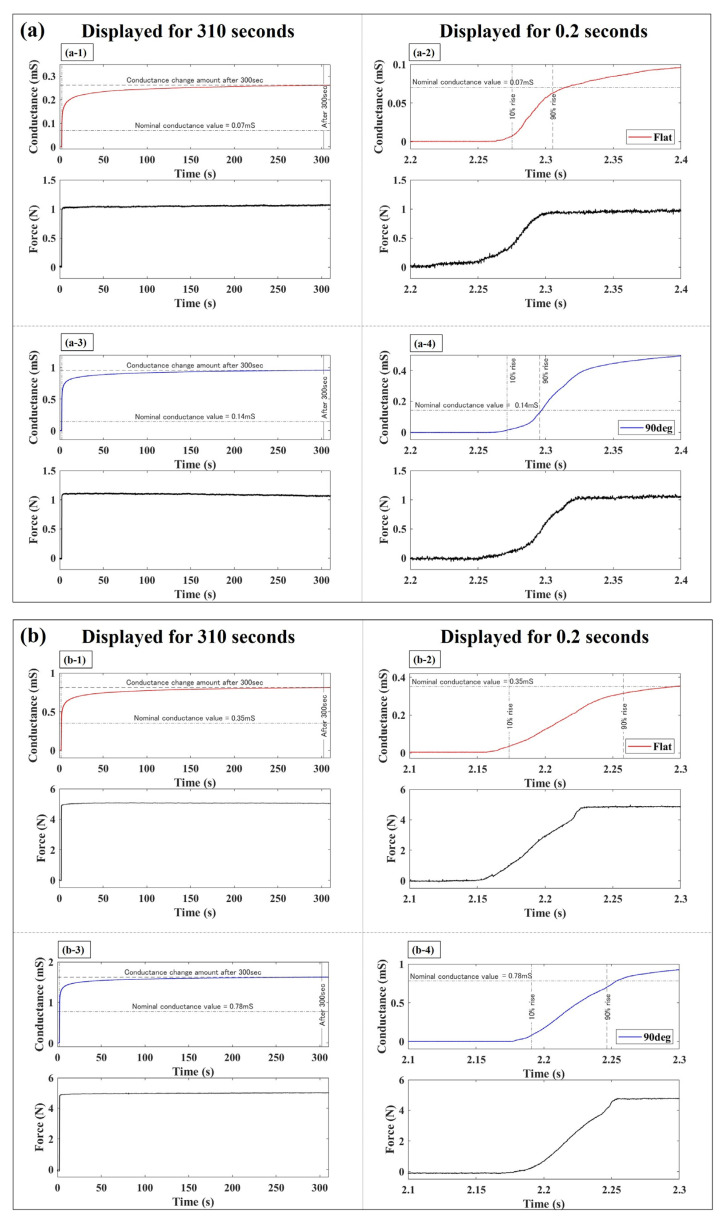

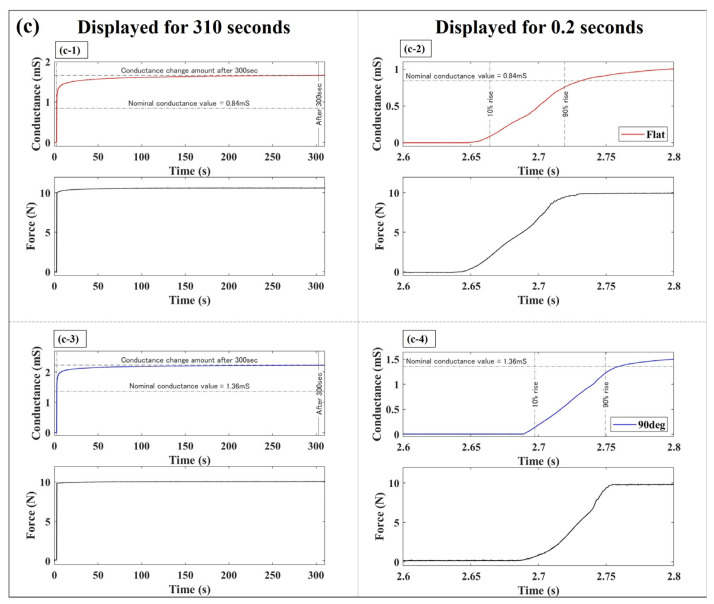
Comparison of elastomeric sensor response to a step-like input: (**a**) 1 N; (**b**) 5 N; (**c**) 10 N (Red: Elastomer sensor response with the flat electrode; Blue: Elastomer sensor response with the 90° electrode; Black: Force sensor response).

Figure 6 depicts the variation in conductance of the elastomer sensor in response to a step-like input applied using a finger robot. The graphs shown on the left side of Figure 6 represent the sensor responses over a duration of 310 s. Notably, the first 0.2 s of sensor responses are shown in the graphs on the right. The waveforms shown in blue and red correspond to the responses of the elastomer sensor when utilizing the 90° electrode and flat electrode, respectively. The measurements obtained using the force sensor are depicted in black. Figure 6a–c showcase the sensor responses under external forces of 1, 5, and 10 N. A dashed line perpendicular to the time axis indicates the moment when 10% of the nominal conductance value is reached, while a solid line denotes the time 300 s thereafter. In Figure 6, the nominal conductance value is indicated by a dashed-dotted line along the horizontal axis of the time axis, while the conductance after applying a load to the elastomer sensor for 300 s is indicated by a broken line. Specifically, the nominal conductance values were 0.07 mS for the flat electrode at 1 N and 0.14 mS for the 90° electrode, 0.35 mS for the flat electrode at 5 N and 0.78 mS for the 90° electrode, 0.84 mS for the flat electrode at 10 N and 1.34 mS for the 90° electrode. The force measurement exhibited a change within 10% of the steady value of the input step-like external force, indicating nearly constant force application. At the time instant of 0.2 s when a step-like input was applied, the step response indicated by the force sensor exceeded 95% of the rated value within 0.08 s for the external forces. Although it was not feasible to exert a step-like external force with a response time shorter than 0.08 s in this experimental environment, we achieved nearly identical response times with high reproducibility. When an external force is applied, as indicated by the force sensor, the conductance changes over time, consistently surpassing the nominal conductance value, regardless of the electrode shape.

**Figure 7:**
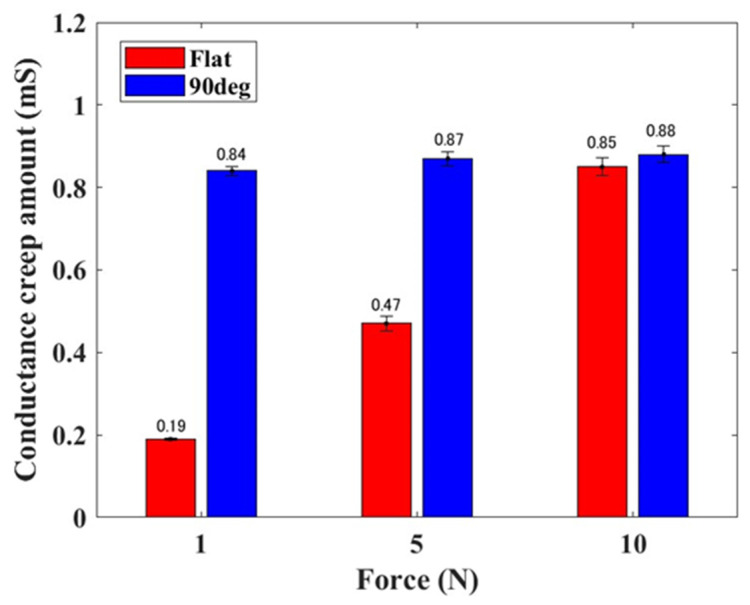
Mean value and standard deviation of conductance creep amount of elastomer sensor in response to step-like inputs.

Figure 7 shows the mean value and standard deviation of the conductance creep amount, derived from five experimental trials. The red and blue bars represent the conductance changes observed with the flat and 90° electrodes, respectively. With the flat electrode, the conductance creep amount increased proportionally to the step-like external force applied to the elastomer sensor. Specifically, with the flat electrode, the conductance increased by 0.19 mS at 1 N, 0.47 mS at 5 N, and 0.85 mS at 10 N relative to the nominal conductance value. Conversely, when utilizing the 90° electrode, the conductance increased by 0.84 mS at 1 N, 0.87 mS at 5 N, and 0.88 mS at 10 N compared to the nominal conductance value. Furthermore, the conductance remained consistent at approximately 0.8 mS~0.9 mS, regardless of the magnitude of the step-like external force applied to the elastomer sensor. Compared to the conductance creep amount with the flat electrode, the use of the 90° electrode exhibited the most significant increase when a step-like external force of 1 N was applied, resulting in a 4.42-fold change in conductance. Generally, when applying a step-like external force of less than 10 N to the elastomer sensor, the conductance creep amount with the 90° electrode is larger than with the flat electrode. This shows a significant difference in the conductance creep amount, particularly evident with 1 and 5 N of step-like external forces.

**Figure 8:**
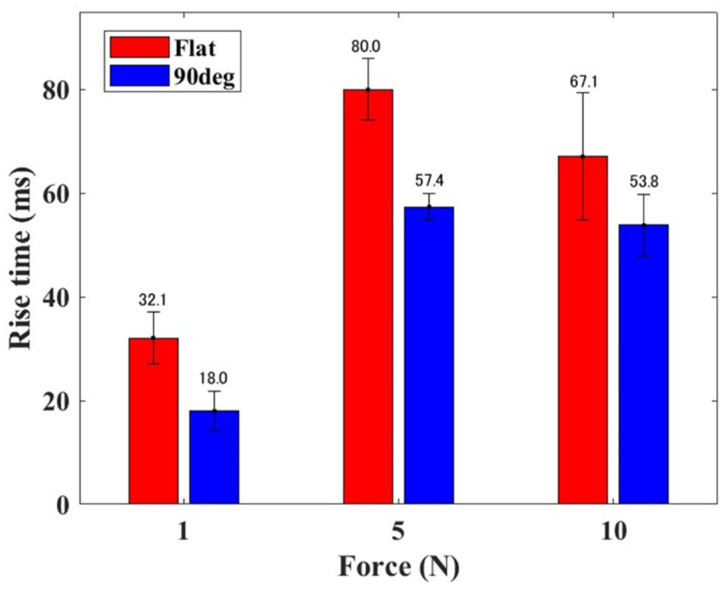
Mean value and standard deviation of the rise time of elastomer sensor in response to step-like inputs.

Figure 8 shows the mean and standard deviation of the rise time of the elastomer sensor response for each external force when subjected to a step-like input. It is evident that, regardless of the magnitude of the step-like external force, the rise time is shorter for the 90° electrode than that for the flat electrode. Moreover, the rise time reached its peak value when a step-like external force of 5 N was applied. When employing the flat electrode, the rise times during the application of step-like external forces applied at 1, 5, and 10 N were 32.1, 80, and 67.1 msec, respectively. The rise times for the 90° electrode under similar step-like external forces applied at 1, 5, and 10 N were 18.0, 57.4, and 53.8 msec, respectively. These findings show that, in comparison with the flat electrode, the rise time is generally shorter when using the 90° electrode.

#### 3.3.2. Sensor Response Evaluation by Periodic Impulse-like Inputs

**Figure 9:**
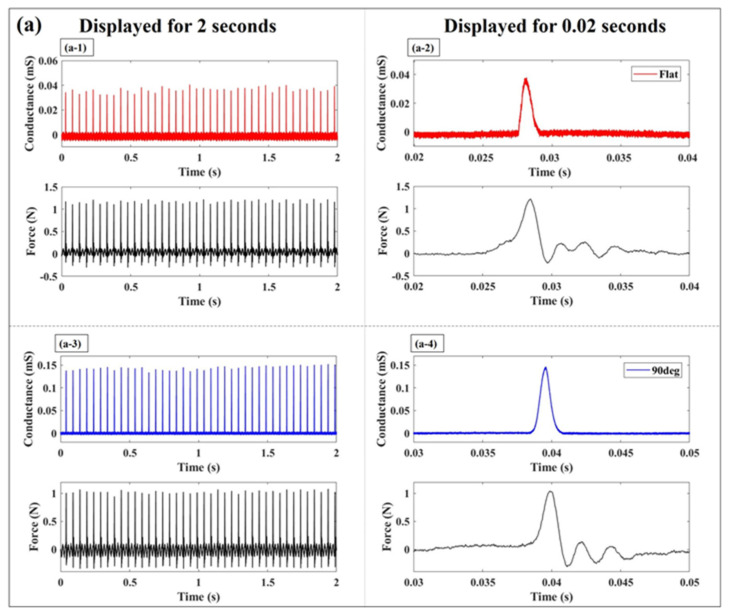

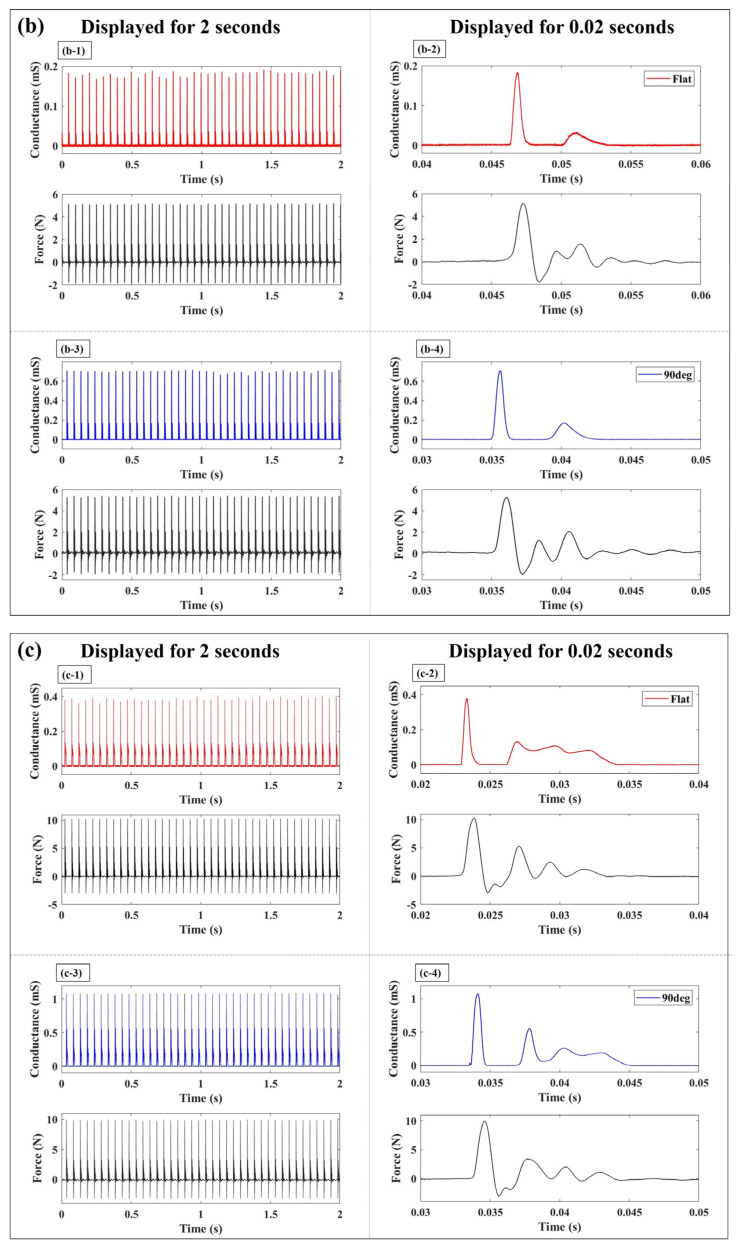
Comparison of elastomeric sensor response to periodic impulse-like inputs: (**a**) 1 N; (**b**) 5 N; (**c**) 10 N (Red: Elastomer sensor response with the flat electrode; Blue: Elastomer sensor response with the 90° electrode; Black: Force sensor response).

Figure 9 shows the sensor response to an impulse-like input applied to the elastomer sensor at a frequency of 20 Hz. The blue waveform represents the response of the elastomer sensor when the 90° electrode was used, while the red waveform depicts the response with the flat electrode. The black waveform corresponds to the response of the force sensor. The left graph shows the sensor response when an impulse-like input is applied for 2 s at a frequency of 20 Hz. Notably, regardless of the electrode or the magnitude of the impulse-like external force, the elastomer sensor consistently detects the external force with an impulse-like input at a frequency of 20 Hz. The waveform on the right displays the sensor response during the first impulse-like input depicted in the left graph, shown at 0.02 s intervals. Upon closer examination of the force sensor waveform during the application of an impulse-like input, it is evident that the force sensor’s response rapidly rises within 4 milliseconds, reaches a peak value, and then declines. However, precise control over the desired maximum magnitude of the impulse-like input is challenging when using the shaker equipment. Particularly, during a 1 N impulse-like input test, a force that was up to 30% greater than 1 N was applied. However, achieving greater precision in experimentation was not feasible. The sensor response waveforms shown in Figure 9b,c will be our focus at this point. Upon applying an impulse-like input, both the elastomer and force sensors exhibited changes, as evidenced by the sensor waveforms in Figure 9(b-2,b-4,c-2,c-4). Notably, the response waveforms continued to fluctuate even after reaching their maximum values for both sensor responses. Following the attainment of the maximum force from the impulse-like external force, the load was released, and a response waveform was obtained upon the reformation of contact with a force smaller than the maximum load. In this experimental setup, there exists a possibility that the shaker indenter rebounds and makes contact again with the resin plate of the elastomer sensor after reaching the maximum external force, a phenomenon unavoidable in this context. Various methods of applying an impulse-like external force have been considered, with the shaker used in this experiment proving to yield the most reproducible input. In analyzing the sensor response within this experimental framework, focus was placed only on the segment showing the peak value over time.

Table 1 presents the nominal conductance values under impulse-like input conditions. The first column lists the maximum value of the impulse-like external force. The second column describes the nominal conductance value observed with the flat electrode, while the third column illustrates the peak conductance recorded during the application of the impulse-like input. Lastly, the fourth column exhibits the ratio of peak conductance to the nominal conductance value, termed as the peak conductance ratio in this study. Corresponding results for the 90° electrode are provided in Table 1, following the same sequence. The peak conductance typically registers lower than the nominal conductance value for both the flat electrode and the 90° electrode. Additionally, as the magnitude of the impulse-like input increases, the conductance measured by the elastomer sensor tends to diminish relative to the nominal conductance value. Specifically regarding the peak conductance ratio with the flat electrode, it is found that upon application of a 1N impulse-like input, conductance value is 57% of the nominal conductance value. Furthermore, when subjected to a 10 N impulse-like input, the peak conductance ratio hits its lowest point, resulting in a conductance of 45%. Similarly, with the 90° electrode, a 7% higher conductance is observed when the external force is 1 N compared to the nominal conductance value. Furthermore, upon applying a 10 N impulse-like input, the peak conductance ratio reaches 79%, surpassing that of the flat electrode. Additionally, the eighth column of Table 1 highlights the amplification of the peak conductance value obtained with the 90° electrode relative to that with the flat electrode when an impulse-like input is applied to the elastomer sensor. Specifically, when external forces of 1, 5, and 10 N were applied, the results were 3.75, 3.88, and 2.84 times, respectively. Notably, these results differ from the magnification of conductance obtained in the quasi-static characteristic evaluation.

Based on the aforementioned results, it is evident that the conductance of the elastomer sensor in response to instantaneous inputs, like impulse inputs, significantly differs from that observed in the evaluation of quasi-static characteristics. Table 2 presents the key results of this section, providing a comprehensive overview of the characteristic evaluation conducted in this study.

## 4. Discussion

**Figure 10:**
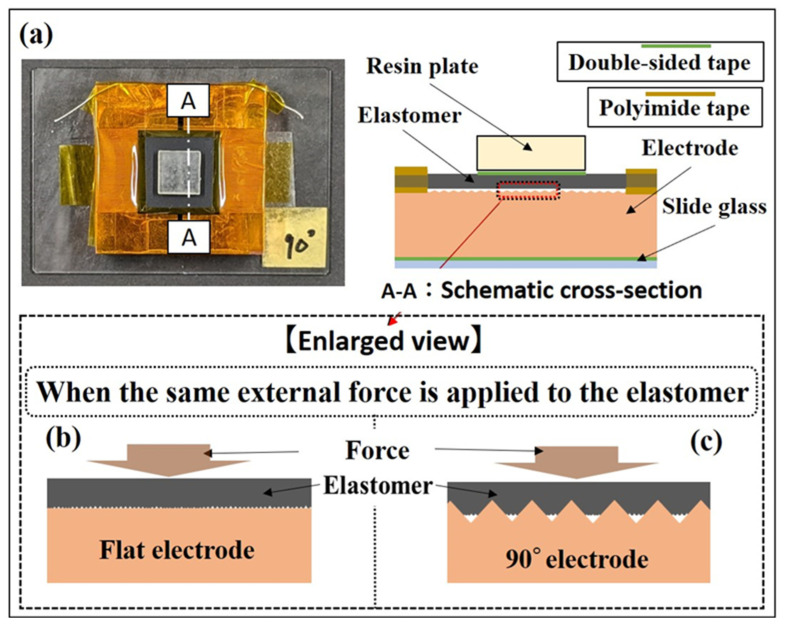
Hypothesis of improved conductivity between elastomer and electrode: (**a**) Position of reference cross-section A-A and schematic cross section of cross section A-A in elastomer sensor; (**b**) Schematic diagram of contact state between elastomer and flat electrode; (**c**) Schematic diagram of contact state between elastomer and 90° electrode.

In this study, we observed enhancements in the quasi-static and dynamic characteristics of the sensor response when utilizing a 90° electrode. One potential explanation for this outcome is that the use of the 90° electrode likely led to an expansion of the contact area between the elastomer and electrode, along with an increase in local contact pressure under the same external force. We hypothesize that these factors contributed to improving the conductivity between the elastomer and electrode. Previous analysis of the conductive elastomer used in this study revealed that its intrinsic conductance remained high even without any applied load. Moreover, when an external force is applied to the elastomer, the contact area between the electrode and elastomer expands, substantially influencing the sensor response [40]. We hypothesize that when the electrode with triangular wave concavo-convex surfaces is used on an elastomer with such characteristics, a deformation of the elastomer as shown in Figure 10 occurs. Figure 10a shows the position of cross section A-A that you would like to refer to in the elastomer sensor, and the schematic cross section of cross section A-A. Focusing on the position indicated by the red dotted line frame in Figure 10a, Figure 10b,c are schematic diagrams when the position of the red dotted line frame in Figure 10a is enlarged. By using the triangular wave concavo-convex surface electrode in conjunction with the elastomer’s inherent properties, it becomes feasible to locally apply high contact pressure on the elastomer. This is attributed to the sharpness of the electrode surface upon the application of the same external force. Such heightened local contact pressure may promote elastomer deformation, thereby creating a more conductive surface area compared to that with the flat electrode. An evaluation of the optimal electrode shape is shown in Figure 4a. Interestingly, the electrode with the largest apparent surface area was the 60° electrode, boasting twice the surface area of the flat electrode. However, despite the 90° electrode having a surface area only 1.41 times higher than that of the flat electrode, it demonstrated superior sensor response throughout the entire process when subjected to 30 N of external force. Conversely, with the 120° electrode, higher conductance values were obtained than with the flat electrode when an external force exceeded 15 N, but lower conductance values were observed compared to the flat electrode. These results indicate that merely increasing the apparent surface area (via concave-convex on the electrode surface) may not necessarily improve sensor response. Therefore, besides altering the apparent surface area of the electrode surface, the electrode’s contact shape, suitable for promoting elastomer deformation, may affect sensor characteristics. In this study, the utilization of a 90° electrode potentially facilitated achieving the highest conductance value. In this study, we were unable to elucidate the principle behind the improved electrical conductivity between the elastomer and the electrode due to the triangular wave concavo-convex surfaces. In addition, we created triangular wave concavo-convex surface with three types of tip angles in the electrode shape, but we were unable to clarify which part of the electrode shape parameters, such as the other tip angles and the height of the convexities, has a dominant influence on the sensor response. Our future challenge is to be able to provide this information by improving our electrode manufacturing environment and processing technology. Furthermore, while this study employed a triangular wave, future endeavors will include evaluating sensor characteristics with alternative shapes, including sine wave, and designing electrode shapes that are optimal for improving sensor performance. Our results underscored the effectiveness of altering the electrode surface shape in modifying sensor response. Moreover, it was evident that disparities in the conductive properties of conductive elastomers resulted in variations in sensitivity improvement and linear pressure-sensitive range. Through the design of electrode surface shape, sensor response characteristics suitable for various practical applications can be achieved. Furthermore, leveraging recently reported three-dimensional microelectrode fabrication technologies [41,42] and MEMS [43], there is potential to further enhance the performance of commercially available conductive elastomers by optimizing electrode surface properties.

## 5. Conclusions

In this study, we proposed the modification of surface shape as a potential method to improve the sensor response of commercially available conductive elastomers. OFC electrodes featuring triangular concavo-convex surfaces were fabricated with tip angles of 60°, 90°, and 120° and their quasi-static and dynamic characteristics were evaluated. During the quasi-static characteristic evaluation, employing a high-sensitivity elastomer with a 90° electrode yielded the highest conductance value with increasing external force. Furthermore, sensitivity evaluation up to 10 N of compression revealed that using the 90° electrode improved sensor response sensitivity by 1.83 times compared to the flat electrode. Moreover, dynamic characteristic evaluation confirmed that when subjected to a step-like external force, the sensor response tended to rise faster with the 90° electrode compared to the flat electrode. Furthermore, when subjecting the system to a periodic impulse-like input of 5 N, the most significant disparity was observed in the peak conductance values obtained. Employing the 90° electrode, as opposed to the flat electrode, resulted in a 3.88-fold increase in conductance. Despite these improvements, certain aspects of sensor performance deteriorated when using the 90° electrode. A trend towards heightened variability in sensor response was noted during quasi-static test conditions. Additionally, in evaluating dynamic characteristics, it was found that conductance creep amount increased at applied 1 and 5 N step-like forces. These results suggest that implementing the outcomes of this research could improve the accuracy of machine learning algorithms by making it easier to identify minute changes in force when touching the sensor and differences in the objects in contact. Moreover, improving the sensor response’s rise time may expedite the control speed of robots, thereby improving their performance. Moreover, sensor responses were obtained using flat electrodes and 90° electrodes for two distinct types of conductive elastomers with varying conductive properties. Across both elastomer types, the conductance values recorded with the 90° electrode surpassed those obtained with the flat electrode. These results suggest that electrodes featuring triangular wave concavo-convex surfaces, irrespective of the specific conductive elastomer used, have the potential to improve the responsiveness of elastomer sensors. Such improvements could stimulate greater demand for conductive elastomer sensors using this method.

## Figures and Tables

**Table 1:** Comparison of conductance between nominal conductance value and peak conductance at an impulse-like input.

	Flat Electrode			90° Electrode			
Force (N)	Nominal conductance value (mS)	Peak conductance at impulse-like inputs (mS)	Peak conductance ratio (%)	Nominal conductance value (mS)	Peak conductance at impulse-like inputs (mS)	Peak conductance ratio (%)	Magnification
1	0.07	0.04	57	0.14	0.15	107	3.75
5	0.35	0.183	52	0.78	0.71	91	3.88
10	0.84	0.38	45	1.36	1.08	79	2.84

**Table 2:** Summary of main results of this study.

Characteristic	Comparison Contents	Main Results
Quasi-static	Effects of differences in electrode surface shape on conductance	In the pressure-sensitive range up to 30 N, higher conductance was obtained with the 60° electrode and the 90° electrode compared to the flat electrode.
	Sensitivity up to 10 N when pressurized	Compared to the flat electrode, the sensitivity improved the most with the 90° electrode, increasing by 1.83 times.
	Variability of sensor response	Compared to the flat electrode, the standard deviation of the sensor response increased with the 90° electrode.
	Adaptability of the 90° electrode to elastomers with different conductive properties	For all of the elastomers with different conductive properties used in this experiment, higher conductance was obtained up to 30 N when using the 90° electrode compared to the flat electrode.
Dynamic	Conductance creep amount	Compared to the flat electrode, the 90° electrode increased conductance creep amount.
	Rise time	Compared to the flat electrode, the 90° electrode shortened the rise time.
	Peak conductance by impulse-like input	Compared to the flat electrode, the 90° electrode achieved conductance more than 2.84 times higher.

## Data Availability

The data presented in this study are available on request from the corresponding author.
